# Knowledge and Attitudes of Critical Care Unit Nurses Regarding Diabetic Ketoacidosis: Palestinian Perspective

**DOI:** 10.1155/nrp/9150016

**Published:** 2025-06-25

**Authors:** Ishaq Alskafi, Ahmad Ayed, Ahmad Batran, Ibrahim Aqtam, Moath Abu Ejheisheh, Mohammed AL Bashtawy

**Affiliations:** ^1^Department of Nursing, Faculty of Allied Medical Sciences, Palestine Ahliya University, Hebron, State of Palestine; ^2^Department of Nursing, Arab American University, Jenin, State of Palestine; ^3^Department of Nursing, Ibn Sina College for Health Professions, Nablus University for Vocational and Technical Education, Nablus, State of Palestine; ^4^Department of Nursing, Al al-Bayt University, Al-Mafraq, Jordan

**Keywords:** attitudes, critical care unit, diabetic ketoacidosis, knowledge

## Abstract

**Introduction:** Diabetic ketoacidosis (DKA) is an acute, progressive, and life-threatening complication of uncontrolled diabetes mellitus requiring immediate and aggressive intervention. If not treated appropriately, DKA can be fatal. Nurses' knowledge and attitudes in critical care units are crucial for providing quality care to minimize the mortality, morbidity, and complications associated with DKA.

**Objectives:** This study assessed the knowledge and attitudes of critical care unit nurses toward DKA in the Southern West Bank hospitals.

**Methods:** A cross-sectional study was conducted with a convenience sample of 178 nurses working in critical care units in Southern West Bank hospitals during February and March 2024. A self-administered questionnaire, developed by the researchers, assessed nurses' knowledge and attitudes toward DKA. The survey included 18 knowledge-based questions covering causes, symptoms, diagnostics, and management, as well as 15 attitude-based items rated on a five-point Likert scale. Data analysis was performed using SPSS Version 23, employing descriptive statistics, *t*-tests, and one-way ANOVA to assess differences between groups.

**Results:** The analysis indicated that the majority of nurses, 109 (61.2%), had a low level of knowledge about the management of DKA. In addition, more than half of the nurses, 96 (53.9%), exhibited a fair attitude toward the management of DKA. There was a significant difference between nurses' knowledge about DKA management and the age of the participants (*p* < 0.05). However, no significant difference was found between nurses' attitudes toward DKA management and their professional characteristics (*p* > 0.05).

**Conclusion:** Findings highlight the need for targeted educational programs to improve nurses' knowledge and attitudes toward DKA management. Implementing standardized training, ensuring access to updated clinical guidelines, and integrating DKA management into continuous professional development can enhance patient outcomes. Future research should explore institutional barriers and effective interventions for improving nurses' competencies in DKA care.

## 1. Introduction

Diabetes mellitus (DM) can lead to diabetic ketoacidosis (DKA), a serious and potentially fatal condition requiring immediate medical intervention [1]. DKA demands aggressive treatment in critical care units, including insulin infusion, intravenous fluids, potassium replacement, and laboratory tests to assess acidosis severity [2]. The condition is marked by decreased circulating insulin, increased blood glucose, and electrolyte imbalances, particularly hyponatremia, leading to dehydration [3]. Approximately 20% of people receive a DM diagnosis due to DKA, with appropriate management reducing mortality to < 5% [4]. Symptoms include polyuria, polydipsia, weight loss, fatigue, dyspnea, vomiting, abdominal discomfort, and polyphagia. Regional prevalence varies (19.5%–43.8%), with higher rates in younger children and Type 1 diabetes patients [4]. In Palestine, the incidence was 166.9 per 100,000 in 2021 [5].

Nurses play a critical role by assessing airways and consciousness, monitoring blood glucose and checking for cerebral edema [3], using cardiac monitors for hyperkalemia, administering IV fluids and correcting electrolytes, and monitoring arterial blood gases (ABGs) and urine output [1]. Despite this, nurses' knowledge and attitudes toward DKA management, especially attitudes, which directly influence clinical decision-making and patient outcomes, remain underexplored in Palestine.

### 1.1. Significance of Study

This study is the first to comprehensively assess Palestinian critical care nurses' knowledge and attitudes toward DKA management. Unlike previous studies that focused on general nursing knowledge of diabetes, this research specifically examines critical care unit nurses, who are responsible for handling severe DKA cases. The findings can help identify gaps in education and training, informing the development of targeted interventions to enhance patient safety and care quality. Moreover, the study's implications extend beyond individual competency; it provides evidence for policymakers and healthcare institutions to improve training programs, establish standardized DKA protocols, and optimize resource allocation for diabetes-related emergencies. Understanding nurses' attitudes toward DKA management is particularly significant as positive attitudes can influence clinical decision-making, patient advocacy, and adherence to evidence-based practices, ultimately impacting patient outcomes in critical care settings.

Therefore, this study aims to assess the level of knowledge and attitudes of critical care unit nurses regarding DKA in Southern West Bank hospitals, identify factors influencing their knowledge and attitudes toward DKA management, and provide recommendations for improving nursing education and training on DKA.

## 2. Methods

This study used a cross-sectional design with a convenience sample of 178 nurses working in critical care units across hospitals in the Southern West Bank, Palestine. Nurses from intensive care units (ICUs) and emergency departments in both governmental and nongovernmental hospitals participated.

A total of 305 nurses work in these units, as reported by the Palestinian Ministry of Health [6] and the Palestinian Nursing Association. The sample size was calculated using Raosoft's online calculator, considering a 0.05 significance level, a 95% confidence interval, and an assumed response distribution of 50%. The calculated sample size was 171, but 191 nurses were recruited to account for potential attrition.

### 2.1. Inclusion and Exclusion Criteria

The inclusion criteria included the following: Nurses with at least 3 months of experience in ICUs or emergency departments, holding at least a diploma in nursing, and providing written consent. The exclusion criteria excluded the following: Nurses with less than 3 months of experience, those not meeting the eligibility criteria, and those who opted out.

### 2.2. Instrumentation

A self-administered questionnaire, developed by the researchers, assessed nurses' knowledge and attitudes toward DKA. The survey included two domains as follows:

1. Knowledge domain (18 items): Adapted from previous studies, covering DKA causes, symptoms, diagnostic tests, and management. Responses were scored as correct (1) or incorrect (0), with total scores categorized based on Bloom's cutoff point into low (< 60%), moderate (60%–79%), and high (80%–100%) knowledge levels.
2. Attitude domain (15 items): Adapted from prior DKA attitude scales, using a 5-point Likert scale (1 = strongly disagree to 5 = strongly agree). Total scores were converted into percentage scores and categorized as poor (< 60%), fair (60%–79%), and good (80%–100%) attitude.

### 2.3. Translation and Validation

The questionnaire, originally in English, was translated into Arabic using a forward–backward method to ensure accuracy. Two bilingual nursing experts performed the initial translation, and a third expert completed the back translation. Minor rewording was made following a pilot study with 15 nurses to improve clarity and cultural relevance.

### 2.4. Reliability Testing

Cronbach's alpha was used to assess internal consistency. The knowledge domain scored 0.86, and the attitude domain scored 0.83, indicating good reliability for both.

### 2.5. Data Collection Procedure

After obtaining ethical approval and hospital permissions, the researchers met with head nurses to explain the study's purpose and distribute the questionnaires. Nurses voluntarily completed the survey during their shifts, taking approximately 20–25 min.

### 2.6. Statistical Analysis

Data were analyzed using SPSS Version 23. Descriptive statistics (frequencies, percentages, mean, and standard deviation) summarized the data. Independent *t*-tests were used to compare knowledge and attitude scores by age, gender (male vs. female), and training status (trained vs. untrained in DKA management). One-way ANOVA was applied to compare education levels and ICU/emergency experience. A *p* value of < 0.05 was considered statistically significant.

### 2.7. Ethical Considerations

Ethical approval was obtained from the Institutional Review Board of Palestine Ahliya University (Project number: CAMS/CCNA/6/124). Written informed consent was collected from all participants, ensuring voluntary participation, anonymity, and confidentiality. The study posed no risks to participants' well-being.

## 3. Results

In the current study, 178 out of 191 nurses participated, resulting in a robust response rate of 93.2%. The analysis of participant demographics revealed that the average age of the nurses was 29.7 years (SD = 5.9), ranging from 22 to 53 years. A significant portion of the participants, 109 (61.2%), were male, and the majority, 127 (71.3%), held bachelor's degrees.

Regarding professional characteristics, a substantial number of nurses, and 127 (71.3%), held the position of staff nurse. In terms of experience, 88 (49.4%) reported having less than 5 years of overall nursing experience, while 129 (72.5%) had less than 5 years of experience specifically in ICU or emergency departments. More than half of the participants, 105 (59.0%), indicated that they had received previous training, seminars, or workshops, and 86 (48.3%) specifically reported training related to diabetes management, as summarized in Table 1.

The findings from the analysis revealed that a significant majority of nurses, 109 (61.2%), demonstrated a low level of knowledge regarding the management of DKA. In addition, the analysis indicated that more than half of the nurses, specifically 96 (53.9%), exhibited a fair level of attitude toward the management of DKA. These results are detailed further in Table 2.

The results indicated a significant difference specifically related to the age of the participants (*p* < 0.05), as detailed in Table 3.

Also, the results indicated that there were no significant differences found between nurses' knowledge levels regarding DKA management and their professional characteristics (*p* > 0.05), as presented in Table 4.

The findings indicated that there were no significant differences observed between nurses' attitudes about DKA management and their demographic characteristics (*p* > 0.05), as detailed in Table 5.

The results indicated that there were no significant differences found between nurses' attitudes toward DKA management and their professional characteristics (*p* > 0.05), as presented in Table 6.

## 4. Discussion

The current study indicated a lack of knowledge among nurses regarding the management of DKA. Several factors contribute to this result, including limited access to continuous education programs specifically addressing DKA management, insufficient clinical exposure to DKA cases, and outdated or inconsistent guidelines within healthcare institutions. In addition, high patient-to-nurse ratios and workload pressures may hinder nurses' ability to stay updated with current practices. The absence of regular competency assessments and limited training resources can further exacerbate knowledge deficits in this critical area of care. Addressing these factors through targeted educational initiatives and policy changes could improve nurses' competency in managing DKA effectively [7, 8]. These findings align with previous studies, such as Ali et al.'s [9], which also reported inadequate knowledge levels among nurses regarding DKA care. Similarly, research by Abdelrahman et al. [10] and Allawi and Ahmed [11] underscored deficiencies in nurses' knowledge about DKA management across different settings, highlighting common challenges such as staff shortages and limited training opportunities.

Regarding attitudes, the investigation found that more than half of the nurses displayed a fair attitude toward managing DKA. This finding is significant as it indicates that healthcare professionals are capable of managing severe conditions such as DKA, which is crucial for ensuring optimal patient care and outcomes within Palestine's healthcare system. Addressing knowledge gaps through targeted training programs and improving resource allocation could potentially enhance both knowledge and attitudes among nurses, thereby improving patient care quality in managing DKA and similar critical conditions [1, 12].

Several studies support the finding that nurses demonstrate a positive or fair attitude toward the management of DKA, despite challenges in their knowledge base. For example, Mekky et al. [8] found that nurses in critical care units showed a positive approach toward managing complex conditions, including DKA, although their knowledge was not always up-to-date. Similarly, Abdelrahman et al. [10] noted that nurses working in emergency departments displayed a fair attitude toward DKA management, which aligned with their strong willingness to improve care through education and training. Moreover, Shaker et al. [7] observed a similar pattern in ICU nurses, where positive attitudes were evident despite some deficiencies in knowledge and hands-on experience with DKA cases. These findings indicate that, even when knowledge gaps exist, nurses often recognize the significance of managing such conditions and are motivated to deliver the best care possible.

In contrast, some studies suggest that nurses' attitudes toward managing DKA might be less optimistic or even negative, primarily due to various environmental and educational barriers. Ali et al. [9] found that, while nurses in critical care settings had a fair attitude toward DKA management, their attitudes were significantly influenced by institutional factors such as lack of resources and insufficient training programs. Nurses in this study reported feeling less confident in managing DKA, which negatively impacted their attitudes toward the condition.

The current study indicated a significant difference between age and knowledge of DKA. This finding suggests that age may play a role in influencing nurses' knowledge levels concerning DKA management, highlighting a potential area for targeted educational interventions or further investigation into age-related factors affecting healthcare knowledge. This contrasts with findings from several other studies. For instance, Mekky et al. [8] conducted a quasiexperimental study at Benha University Hospital involving 70 ICU nurses and found no significant association between age and DKA knowledge. Similarly, Abdelrahman et al. [10] conducted a descriptive study at Assuit University Hospital with 35 emergency department nurses and no significant correlation between age and knowledge level (*p* value = 0.338). In addition, Allawi and Ahmed [11] conducted a descriptive study in Mosul City, Iraq, among 30 nurses in emergency departments and ICUs and found no significant association between age and DKA knowledge. Ali et al. [9] conducted a descriptive study at Ain Shams University with 40 nurses in medical ICUs, while Shaker et al. [7] conducted a quasiexperimental study among 40 ICU nurses at El-Fayoum University Medicine Hospitals. Neither study found a significant association between age and DKA knowledge.

The current study revealed no significant differences between gender and nurses' knowledge or attitudes regarding the management of DKA. This finding is consistent with previous research, which has also reported no significant association between gender and clinical knowledge or attitudes among nurses. For example, studies conducted by Allawi and Ahmed [11], Ali et al. [9], and Abdelrahman et al. [10] support the notion that gender does not inherently influence nurses' competency levels or their perceptions of DKA management.

Also, the current study indicated no significant differences between the level of education and nurses' knowledge or attitudes about DKA. Ali et al. [9] conducted a descriptive exploratory study at Ain Shams University with 40 nurses, where 72.5% had diploma degrees and 10% had bachelor's degrees, yet no significant relationship was found between educational level and DKA knowledge or attitudes. Similarly, Abdelrahman et al. [10] conducted a descriptive study at Assuit University Hospital among 35 critical care unit nurses, with 28.6% having diploma degrees and 8.6% having bachelor's degrees, and no significant association was observed between educational level and DKA knowledge.

In addition, the study indicated no significant differences between nurses' knowledge or attitudes regarding the management of DKA and the job position of the participants. Also, there are no significant differences between nurses' knowledge or attitudes regarding the management of DKA and the total experience of the participants.

Furthermore, the result showed no significant differences between nurses who received training about DKA and nurses' knowledge or attitudes about DKA. Ali et al. [9] conducted a descriptive exploratory study at Ain Shams University with 40 medical ICU nurses, where 25% had received previous training about diabetes and 75% had not. Similarly, Abdelrahman et al. [10] conducted a descriptive study at Assuit University Hospital among 35 critical care unit nurses, reporting that 40% had received one course of previous training about diabetes, 28.6% had received two courses, and 25.7% had received three courses. In addition, Mekky et al. [8] conducted a quasiexperimental study at Benha University Hospital with 70 critical care unit nurses, where only 7.1% had received previous training about diabetes and 92.9% had not. Likewise, Shaker et al. [7] conducted a quasiexperimental study at El-Fayoum University with 40 ICU nurses, where none of the nurses had received previous training about diabetes. Across these studies, there was no significant association found between nurses' participation in training about diabetes and their knowledge or attitudes toward managing DKA. These findings from our Palestinian context align with the international literature, suggesting that current training approaches may need to be restructured to be more effective in improving DKA management competencies among critical care nurses.

### 4.1. Strengths and Limitations of the Study

To our knowledge, this is the first study in West Bank hospitals that assessed nurses' knowledge and attitude about DKA in the critical care unit and included multiple variables about DKA. However, several limitations should be acknowledged. This study's focus on Southern West Bank hospital nurses may restrict its generalization ability to other healthcare settings, using a cross-sectional design and convenience sample methods. However, this study served as a foundation for future studies.

### 4.2. Recommendations

Targeted continuing education programs for critical care unit nurses should be conducted, focusing on evidence-based guidelines to improve the quality of care for DKA patients. This should be supported by further studies on a larger population and sample size to ensure broader applicability. In addition, it is recommended that the Ministry of Health circulate a unified protocol for DKA management across all hospitals, ensuring consistency in care. Finally, enhancing nurses' self-education will empower them with more knowledge, which can help reduce complications and improve patient's outcomes.

## 5. Conclusion

The study confirmed that the majority of nurses had a low level of knowledge, while more than half of them had a fair level of attitude regarding DKA. The current study indicated a statistically significant difference between nurses' knowledge regarding DKA and the age of participants. Furthermore, there were no statistically significant differences between a nurse's knowledge and attitudes and professional characteristics.

## Figures and Tables

**Table 1:** Distribution of sociodemographic and professional characteristics among nurses (*N* = 178).

**Variable**		***N* (%)**

Age	Age (years old) (*M* = 29.7, SD = 5.9, min = 22, max = 53)	
	Less than 30 years	108 (60.7)
	30 years and above	70 (39.3)

Gender	Male	109 (61.2)
	Female	69 (38.8)

Level of education	Diploma	41 (23.0)
	Bachelor's degree	127 (71.3)
	Master's degree and above	10 (5.6)

Job position	Practical nurse	47 (26.4)
	Staff nurse	127 (71.3)
	Head nurse	4 (2.2)

Total experience	Less than 5 years	88 (49.4)
	5–10 years	60 (33.7)
	More than 10 years	30 (16.9)

Experience in the ICU or emergency department	5 years and less	129 (72.5)
	6–10 years	28 (15.7)
	More than 10 years	21 (11.8)

Have you received previous training, seminars, and workshops?	Yes	105 (59.0)
	No	73 (41.0)

Have you received previous training, seminars, and workshops about diabetes?	Yes	86 (48.3)
	No	92 (51.7)

**Table 2:** Description of the “participants' knowledge and attitudes about the management of diabetic ketoacidosis” (*N* = 178).

Variable	*N* (%)	*M* (SD)
Knowledge		54.6 (14.1)
“Low-level knowledge”	109 (61.2)	
“Moderate-level knowledge”	65 (36.5)	
“High-level knowledge”	4 (2.2)	
Attitude		71.3 (13.7)
Poor level	32 (18.0)	
Fair level	96 (53.9)	
Good level	50 (28.1)	

*Note: N* = sample; % = percentage.

**Table 3:** Differences between knowledge mean and demographic characteristics (*N* = 178).

Variable	*N*	*M*	SD	Statistical *t*-test	*p* value
Age					
Less than 30 years	108	56.7	14.0	*t* = 2.606	0.010
30 years and above	70	51.2	13.6		
Gender					
Male	109	54.5	15.0	*t* = 0.024	0.981
Female	69	54.6	12.6		
Level of education					
Diploma	41	57.7	13.8		
Bachelor's degree	127	53.1	14.2	*F* = 2.703	0.070
Master's degree and above	10	60.6	10.6		

*Note: t* = Student's *t*-test; *F* = one-way ANOVA.

Abbreviations: *M* = mean; SD = standard deviation.

**Table 4:** Differences between knowledge mean and professional work characteristics (*N* = 178).

Variable	*N*	*M*	SD	Statistical *t*-test	*p* value
Job position					
Practical nurse	47	53.8	14.7	*F* = 0.141	0.869
Staff nurse	127	54.8	14.1		
Head nurse	4	56.9	8.3		
Total experience					
Less than 5 years	88	56.6	14.5	*F* = 1.926	0.149
5–10 years	60	53.1	13.6		
More than 10 years	30	51.5	13.4		
Experience in the ICU or emergency department					
5 years and less	129	55.3	14.0	*F* = 0.648	0.524
6–10 years	28	52.8	13.9		
More than 10 years	21	52.4	15.5		
Have you received previous training, seminars, and workshops?					
Yes	105	55.3	14.9	*t* = 0.832	0.406
No	73	53.5	12.9		
Have you received previous training, seminars, and workshops about diabetes?					
Yes	86	54.6	14.2	*t* = 0.027	0.978
No	92	54.6	14.1		

*Note: t* = Student's *t*-test; *F* = one-way ANOVA.

Abbreviations: *M* = mean; SD = standard deviation.

**Table 5:** Differences between attitude mean and demographic characteristics (*N* = 178).

Variable	*N*	*M*	SD	Statistical *t*-test	*p* value
Age					
Less than 30 years	108	72.3	12.1	*t* = 1.171	0.243
30 years and above	70	69.8	16.0		
Gender					
Male	109	71.2	13.3	*t* = −0.117	0.907
Female	69	71.5	14.6		
Level of education					
Diploma	41	72.7	14.4		
Bachelor's degree	127	70.4	13.2	*F* = 1.237	0.293
Master's degree and above	10	76.7	17.2		

*Note: t* = Student's *t*-test; *F* = one-way ANOVA.

Abbreviations: *M* = mean; SD = standard deviation.

**Table 6:** Differences between attitude mean and professional work characteristics (*N* = 178).

Variable	*N*	*M*	SD	Statistical *t*-test	*p* value
Job position					
Practical nurse	47	68.8	13.9	*F* = 1.122	0.328
Staff nurse	127	72.2	13.5		
Head nurse	4	73.3	21.9		
Total experience					
Less than 5 years	88	72.9	12.1	*F* = 1.283	0.280
5–10 years	60	69.3	15.4		
More than 10 years	30	70.8	14.9		
Experience in the ICU or emergency department					
5 years and less	129	71.7	13.0		
6–10 years	28	70.4	17.2	*F* = 0.163	0.850
More than 10 years	21	70.3	13.7		
Have you received previous training, seminars, and workshops?					
Yes	73	53.5	12.9	*t* = 1.602	0.111
No	105	70.0	13.6		
Have you received previous training, seminars, and workshops about diabetes?					
Yes	86	69.9	14.2	*t* = 1.349	0.170
No	92	72.7	13.2		

*Note: t* = Student's *t*-test; *F* = one-way ANOVA.

Abbreviations: *M* = mean; SD = standard deviation.

## Data Availability

The data that support the findings of this study are available on request from the corresponding author. The data are not publicly available due to privacy or ethical restrictions.
